# Patterns of bruising in preschool children with inherited bleeding disorders: a longitudinal study

**DOI:** 10.1136/archdischild-2015-310196

**Published:** 2016-07-22

**Authors:** Peter W Collins, Melinda Hamilton, Frank D Dunstan, Sabine Maguire, Diane E Nuttall, Ri Liesner, Angela E Thomas, John Hanley, Elizabeth Chalmers, Victor Blanchette, Alison M Kemp

**Affiliations:** 1 Department of Haematology, Institute of Infection and Immunity, School of Medicine Cardiff University, Cardiff, UK; 2 Department of Haematology, Ysbyty Gwynedd, Bangor, UK; 3 Institute of Primary Care and Public Health, School of Medicine Cardiff University, UK; 4 Department of Haematology, Great Ormond Street Hospital, London, UK; 5 Department of Haematology, Royal Hospital for Sick Children, Edinburgh, UK; 6 Department of Haematology, Royal Victoria Infirmary, Newcastle, UK; 7 Department of Haematology, Royal Hospital for Sick Children, Glasgow, UK; 8 Department of Paediatrics, University of Toronto, Toronto, Canada; 9 Division of Haematology/Oncology, The Hospital for Sick Children, Toronto, Canada

**Keywords:** Inherited bleeding disorders, Bruising, Mobility Stage, Haemophilia

## Abstract

**Objective:**

The extent that inherited bleeding disorders affect; number, size and location of bruises in young children <6 years.

**Design:**

Prospective, longitudinal, observational study.

**Setting:**

Community.

**Patients:**

105 children with bleeding disorders, were compared with 328 without a bleeding disorder and classified by mobility: premobile (non-rolling/rolling over/sitting), early mobile (crawling/cruising) and walking and by disease severity: severe bleeding disorder factor VIII/IX/XI <1 IU/dL or type 3 von Willebrand disease.

**Interventions:**

Number, size and location of bruises recorded in each child weekly for up to 12 weeks.

**Outcomes:**

The interventions were compared between children with severe and mild/moderate bleeding disorders and those without bleeding disorders. Multiple collections for individual children were analysed by multilevel modelling.

**Results:**

Children with bleeding disorders had more and larger bruises, especially when premobile. Compared with premobile children without a bleeding disorder; the modelled ratio of means (95% CI) for number of bruises/collection was 31.82 (8.39 to 65.42) for severe bleeding disorders and 5.15 (1.23 to 11.17) for mild/moderate, and was 1.81 (1.13 to 2.23) for size of bruises. Children with bleeding disorders rarely had bruises on the ears, neck, cheeks, eyes or genitalia.

**Conclusions:**

Children with bleeding disorder have more and larger bruises at all developmental stages. The differences were greatest in premobile children. In this age group for children with unexplained bruising, it is essential that coagulation studies are done early to avoid the erroneous diagnosis of physical abuse when the child actually has a serious bleeding disorder, however a blood test compatible with a mild/moderate bleeding disorder cannot be assumed to be the cause of bruising.

**What is already known on this topic?**

- Bruising in children without an inherited bleeding disorder increases with mobility, but is very uncommon in premobile children in the absence of physical abuse.
- In children without an inherited bleeding disorder, bruises are rare on the ears, eyes, neck and genitalia at all mobility stages.
- There are no data available on the pattern of bruising to be expected in young children with an inherited bleeding disorder.

**What this study adds?**

- Children with severe inherited bleeding disorders have more, and larger, bruises than children without an inherited bleeding disorder, especially when premobile.
- The location of bruises on children with an inherited bleeding disorder differs from those without an inherited bleeding disorder.
- At all mobility stages, for children with or without an inherited bleeding disorder, bruises were rare on the ears, neck, cheeks, eyes or genitalia.

## Introduction

Bruising in children may result from normal activity, accidental trauma, physical abuse or haemostatic impairment.^1–4^ Studies have documented the number and pattern of bruises in physically abused and healthy children.^5–7^ The number of bruises that a child has increases with developmental stage^7–10^ and certain sites are bruised more commonly in children who have been physically abused.^9^ There are few studies of the pattern of bruising in children with inherited bleeding disorders who are assumed to have more and larger bruises. Bruises >1 cm contribute to higher paediatric bleeding scores in these children.^11–13^

Young children are the most vulnerable for both undiagnosed bleeding disorders and physical abuse. Diagnosis is based on an assessment of bruises in the context of the proposed history and laboratory investigations (http://www.rcpch.ac.uk/news/rcpch-launches-2nd-edition-child-protection-companion). The interpretation of laboratory haemostatic tests may be difficult.^1–4^ ^14^ If a mild laboratory abnormality is assumed to be the cause of bruising then abuse may go unrecognised but, if a bleeding disorder is missed, a family may be inappropriately accused of abuse. Interpretation of clinical findings may differ between paediatricians and haematologists^15^ and both conditions may coexist.^16^ This study aims to characterise bruising in children with bleeding disorders at different developmental stages, in comparison to children without bleeding disorders.^7^ The data set generated will help paediatricians and haematologists to assess bruising in young children and give an indication of the pattern of bruising to be expected from daily activities of children with a bleeding disorder.

## Methods

This prospective, longitudinal study recruited children age <6 years from six haemophilia centres. Children without bleeding disorders were from well-baby clinics, hospital outpatient clinics, and mother and baby groups in South Wales and have been described previously.^7^ Bleeding disorders were recorded as haemophilia, factor XI deficiency, von Willebrand disease (VWD) or platelet disorders; other bleeding disorders were not recruited. Severe bleeding disorders were defined as factor VIII/IX/XI <1 IU/dL or type 3 VWD. Mild/moderate bleeding disorders were defined as factor VIII/IX/XI ≥1 IU/dL, type 1 and type 2 VWD. Children with mild and moderate bleeding disorder were not analysed separately due to an insufficient number of subjects. Children were subdivided according to prophylactic replacement therapy use.

Parents gave written consent and were trained to record bruise number, location and size on a body map. Developmental stages were defined as premobile (non-rolling, rolling over and sitting), early mobile (crawling or cruising) and walking. Size was the maximum dimension measured with a tape measure. These data ‘collections’ were recorded once weekly for up to 12 weeks. A child could contribute up to 12 weekly collections at each developmental stage, subsequent collections were excluded. In a validation exercise, 40 data collections from 40 non-bleeding disorder children were performed independently by both a carer and researcher and compared.

Bruises were recorded in 38 locations. These were grouped into 18 locations, combining locations on both sides of the body where laterality was considered unimportant or where bruises were rare. The ‘facial-T’ was defined as forehead, nose, lips or chin.^17^ The ‘head’ was the area within the hairline. ‘Front trunk’ included chest and abdomen. ‘Rear trunk’ included the back above buttocks. Bruises from immunisations or venepuncture were excluded. Total bruise size was the sum of the maximum diameters of all bruises in a collection.

### Statistical analysis

Data were summarised using mean and SD or count and proportions and compared between children with and without bleeding disorders. Longitudinal analysis was performed on the number and size of bruises using multilevel modelling, with collections nested within children, to allow comparisons between groups of children with different numbers of collections; non-bleeding disorder children were the reference group.

For bruise number, a model with a Poisson distribution and log link function was used. Comparisons were expressed as ratio of means. For bruises >1 cm a logistic model was employed, leading to an OR for a bruise >1 cm. For bruise size a lognormal distribution was used, and a γ distribution for total bruise size, with a better fit arising from adding one to total size; distributions were chosen following explorations of the distributions of the data. From the multilevel modelling, estimates were derived of the percentage of variation in bruise counts across collections due to differences between children, as opposed to variation in the counts longitudinally within children.

The analysis of bruise size was based only on collections in which all sizes were recorded, in case there was a tendency to record sizes in certain locations, or of certain sizes, preferentially over others. Analysis was performed using Stata V.13.

## Results

The study enrolled 105 children with bleeding disorders, mean age (range) 2.6 (0.1–5.8) years and 328 children without a bleeding disorder mean age (range) 1.6 (0–5.8) years.^7^ There were 58 children with severe and 47 with mild/moderate bleeding disorders. The mild/moderate group had a median (IQR) (range) factor level of 23 (11–30) (3–35 IU/dL). Two patients with platelet function disorders are reported individually. Thus 103 BD children are included in the main analysis (table 1).

**Table 1:** Characteristics of children with and without inherited bleeding disorders

	Non-bleeding disorder	Severe bleeding disorder		Mild/ moderate bleeding disorder
Subjects	328	57		46
Age at enrolment in years Mean (SD)	1.58 (1.45)	2.66 (1.66)		2.43 (1.78)
Gender (M/F)	145 (46%)/168 (54%)	56 (98%)/1(2%)		31 (69%)/14 (31%)
Missing data	15	0		1
Diagnosis		**No prophylaxis**	** Prophylaxis**	
Haemophilia*		27 (47%)	24 (42%)	23 (50%)
VWD	NA	6 (11%)	0 (0%)	23 (50%)
Collections†		**No prophylaxis**	** Prophylaxis**	
Pre mobile	1010 (39%)	64 (18%)	0 (0%)	59 (11%)
Early mobile	478 (19%)	64 (18%)	12 (5%)	70 (13%)
Walking	1082 (42%)	229 (64%)	255 (95%)	393 (75%)
Total	2570	357	267	522

The children with a bleeding disorder (BD) had mean (range) 11 (1–36) collections/child. At the first collection for the bleeding disorder group, 15 children were premobile, 10 early mobile and 78 walking. At first collection for the non-bleeding disorder group 133 children were premobile, 43 early mobile and 152 walking.

*The haemophilia group included 61 with haemophilia A, 10 with haemophilia B, 3 with factor XI deficiency. All children with factor XI deficiency were in the non-severe group.

†Some subjects contributed collections in more than one developmental stage; of the 431 children (excluding the 2 with platelet disorders) 356 remained in a single developmental stage, 65 crossed two and 10 crossed three stages. Some children moved from on demand to prophylaxis. The two patients with platelet function disorders have not been included.

NA, not applicable; VWD, von Willebrand disease.

### Validation

The 40 data collections in the validation cohort had a mean (SD) 2.35 (2.2) bruises/child, range 0–7. There was complete agreement between carers and researcher regarding bruise number and location, the mean difference between the size measurement was <1 mm.

### Number of bruises

There were 5613 bruises recorded from 1146 collections in 103 children with bleeding disorders, and 3523 bruises from 2570 collections in 328 children without a bleeding disorder.

#### Premobile children

Premobile children with mild/moderate bleeding disorders had the same proportion of collections with at least one bruise as those without bleeding disorders (7%). However, children with severe bleeding disorders had at least one bruise in 52% of collections.

Premobile children with bleeding disorders had a higher mean number of bruises/collection than those without a bleeding disorder. Compared with the non-bleeding disorder group the modelled ratio of means (95% CI) for number of bruises/collection for severe bleeding disorders was 31.82 (8.39 to 65.42) and 5.15 (1.23 to 11.17) for the mild/moderate group (table 2).

**Table 2:** Number of bruises in children with and without inherited bleeding disorders

	Number of bruises per collection Mean (SD)	Number and percentage of collections with at least one bruise	Modelled ratio of mean number of bruises (95% CI)
Premobile			
Non-BD	0.09 (0.35)	68/1010 (7%)	1
Mild/moderate BD	0.19 (0.80)	4/59 (7%)	5.15 (1.23 to 11.17)*
Severe BD	1.06 (1.48)	33/64 (52%)	31.82 (8.39 to 65.42)*
Early mobile			
Non-BD	0.80 (1.19)	218/478 (46%)	1
Mild/moderate BD	2.53 (4.78)	39/70 (56%)	2.96 (1.41 to 4.42)*
Severe BD off prophylaxis	7.81 (7.30)	63/64 (98%)	8.28 (3.34 to 13.53)*
Walking			
Non-BD	2.82 (2.77)	852/1082 (79%)	1
Mild/moderate BD			
All patients	5.28 (5.22)	332/393 (84%)	1.66 (1.20 to 1.98)*
Haemophilia	4.11 (3.30)	174/205 (85%)	NA
VWD	6.57 (6.48)	158/188 (84%)	NA
Severe BD off prophylaxis			
All patients	5.62 (5.83)	208/229 (91%)	2.13 (1.53 to 2.55)*
Haemophilia	4.57 (4.14)	164/184 (89%)	NA
VWD	9.91 (9.03)	44/45 (98%)	NA
Severe BD on prophylaxis			
All patients	5.57 (4.50)	232/255 (91%)	1.80 (1.2 to 2.15)*
Haemophilia	5.69 (4.54)	223/246 (91%)	NA
VWD	2.22 (0.67)	9/9 (100%)	NA

NA is not analysed due to insufficient numbers of collections.

*Indicates a statistically significant difference from children without a bleeding disorder. There were insufficient subjects to investigate VWD separately in the premobile and early mobile groups. Prophylaxis is predominantly used for mobile children with severe bleeding disorders and so can only be reported in the walking group.

BD, bleeding disorder; VWD, von Willebrand disease.

#### Early mobile children

In early mobile children with a severe bleeding disorder, 98% of collections had at least one bruise. Children with both severe and mild/moderate bleeding disorders had more bruises/collection than children without a bleeding disorder, but the difference was less marked than among premobile children (table 2).

#### Walking children

Among walking children, the number of bruises/collection, and the percentage of collections with at least one bruise, were similar for the mild/moderate and severe bleeding disorder groups but more than for children without a bleeding disorder. The differences were less marked than in premobile and early mobile children (table 2).

#### Interpatient and intrapatient variations

Among premobile children without a bleeding disorder, the percentage of variation due to random variation over time, as opposed to differences between children, was 64%; for children with a bleeding disorder of any severity, this was lower at 1%. For early mobile children the corresponding values were 42% and 4%, while for walking children they were 25% and 16%. Thus, the amount of variation due to random variation over time declined among non-bleeding disorder children as developmental stages increased, in direct contrast to those with a bleeding disorder.

#### Haemophilia and VWD and effect of prophylaxis

The number of bruises/collection was similar when comparing VWD with haemophilia, or between those receiving, or not receiving, prophylaxis (table 2).

### Size of bruises

Children with severe bleeding disorders had larger bruises than non-bleeding disorder children at all developmental stages. The modelled means (95% CI) for size of bruises for severe premobile bleeding disorders was 1.81 (1.22 to 2.23) (table 3).

**Table 3:** Size of bruises in children with and without inherited bleeding disorders

	Size of bruise Mean (SD)*	Modelled ratio of bruise size (95% CI)	Number and percentage of bruises >1 cm	Number and percentage of collections with at least 1 bruise >1 cm	Modelled OR for collection with a bruise >1 cm	Number and percentage of collections with at least 5 bruises >1 cm	Total bruise size per collection Mean (SD)	Modelled ratio of total bruise size (95% CI)
Premobile								
Non-BD	0.84 (0.52)	1	11/74 (14.9%)	10/1002 (1.0%)	1	0/1002 (0%)	0.06 (0.32)	1
Mild/moderate BD	1.37 (0.97)	1.51 (0.82 to 2.10)	4/11 (36.4%)	2/59 (3.4%)	5.94 (0.61 to 20.38)	0/59 (0%)	0.26 (1.10)	1.39 (1.16 to 1.53)†
Severe BD	1.31 (1.10)	1.81 (1.22 to 2.23)†	17/51 (33.3%)	14/57 (24.6%)	112.6 (11.99 to 378.3)†	0/57 (0%)	1.15 (2.01)	2.32 (1.92 to 2.57)†
Early mobile								
Non-BD	0.95 (0.86)	1	84/335 (25.1%)	67/451 (14.9%)	1	0/451 (0%)	0.71 (1.30)	1
Mild/moderate BD	1.06 (1.22)	1.22 (0.83 to 1.50)	43/173 (24.9%)	23/68 (33.8%)	3.38 (1.63 to 5.02)†	2/68 (2.9%)	2.71 (4.36)	1.83 (1.30 to 2.21)†
Severe BD off prophylaxis	1.54 (1.21)	1.60 (1.05 to 2.01)†	99/226 (43.8%)	31/41 (75.6%)	40.81 (7.01 to 105.8)†	9/41 (22.0%)	8.51 (8.27)	4.66 (2.96 to 5.95)†
Walking								
Non-BD	0.95 (0.80)	1	671/2871 (23.4%)	413/1027 (40.2%)	1	8/1072 (0.8%)	2.65 (2.96)	1
Mild/moderate BD								
All patients	1.20 (0.91)	1.16 (1.01 to 1.24)†	512/1681 (30.5%)	205/350 (58.6%)	2.87 (1.47 to 4.13)†	24/350 (6.9%)	5.77 (6.64)	1.62 (1.28 to 1.84)†
Haemophilia			212/797 (26.6%)	111/199 (55.8%)			4.49 (4.41)	
VWD			300/884 (33.9%)	94/151 (62.3%)			7.46 (8.48)	
Severe BD off prophylaxis								
All patients	1.43 (1.24)	1.47 (1.27 to 1.59)*	350/992 (35.3%)	116/190 (61.1%)	3.43 (1.52 to 5.32)†	28/190 (14.7%)	7.44 (8.45)	2.17 (1.67 to 2.50)†
Haemophilia	1.51 (1.35)		267/706 (37.8%)	95/160 (59.4%)			6.65 (7.70)	
VWD	1.23 (0.90)		83/286 (29.0%)	21/30 (70.0%)			11.69 (10.87)	
Severe BD on prophylaxis								
All patients	1.49 (1.24)	1.41 (1.23 to 1.52)†	396/1019 (38.9%)	135/191 (61.1%)	5.78 (2.55 to 9.01)†	27/191 (14.1%)	7.97 (7.98)	2.12 (1.63 to 2.44)†
Haemophilia	1.50 (1.25)		393/1001 (39.3%)	132/183 (72.1%)			8.21 (8.07)	
VWD	1.08 (0.46)		3/18 (16.7%)	3/8 (37.5%)			2.44 (0.98	

*The mean size of bruises reported may not reflect the actual bruise size because of variation in measurements and individual subjects contribute multiple collections. The relative size of bruises as described by modelled ratios show more reliable differences between groups because all subjects used the same collection methodology and multiple collections have been taken into account through multilevel modelling. The logistic regression investigated the presence of at least one large bruise or not. The categories were: At least 1 bruise >1 cm versus no bruise >1 cm and the latter included collections with no bruises.

†Indicated a statistically significant difference compared with children without a bleeding disorder. Of the 9136 bruises from both groups of children, size was not recorded for 1100 (12%). The proportion of missing bruise sizes varied little across development stages and bleeding disorder status except for two children in the early mobile group, of whom one had 46 and the other 229 bruises with no recorded sizes across collections and were responsible for the high proportion of missing values in this developmental group. Collections with incomplete data on sizes were excluded, resulting in the removal of 279 collections from 98 subjects on whom 1695 bruises were reported, leaving 3437 collections and 7441 bruises for analysis of size.

BD, bleeding disorder; VWD, von Willebrand disease.

### Bruises larger than 1 cm

Across all developmental stages children with bleeding disorders had more bruises of >1 cm than non-bleeding disorder children. Premobile children without a bleeding disorder had at least one bruise of >1 cm in 1.0% of collections, compared with 3.4% of mild/moderate and 24.6% of severe bleeding disorders. There were no collections with at least five bruises of >1 cm in any premobile group. Premobile children with severe bleeding disorders had more bruises/collection of >1 cm than children without a bleeding disorder (modelled OR 112.6, 95% CI 11.99 to 378.3). Early mobile children with a severe bleeding disorder had at least five bruises of >1 cm in 22% of collections (table 3). Total bruise size per collection at each mobility stage is shown in table 3.

### Location of bruises

#### Premobile children

**Figure 1:**
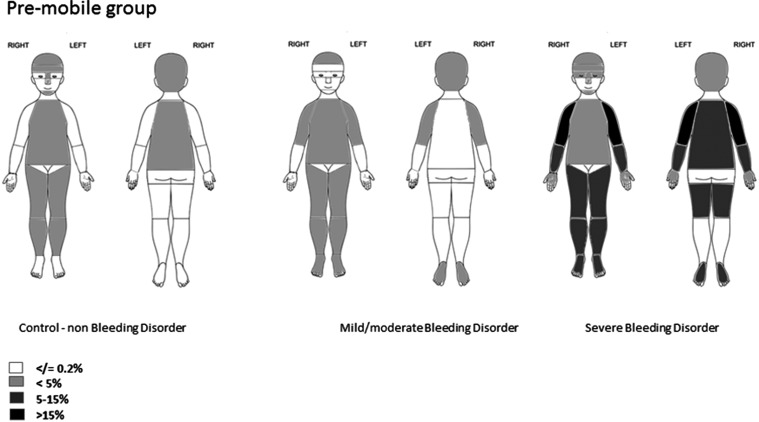
Location of bruises in premobile children. The figure shows the proportion of collections in which there was at least one bruise at the location indicated.

**Figure 2:**
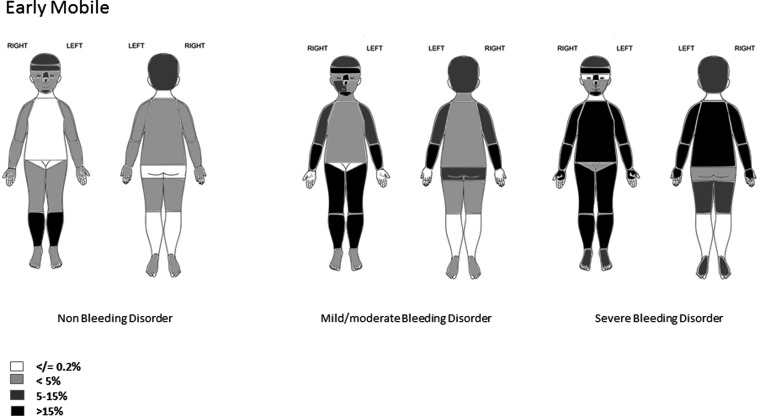
Location of bruises in early mobile children. The figure shows the proportion of collections in which there was at least one bruise at the location indicated.

**Figure 3:**
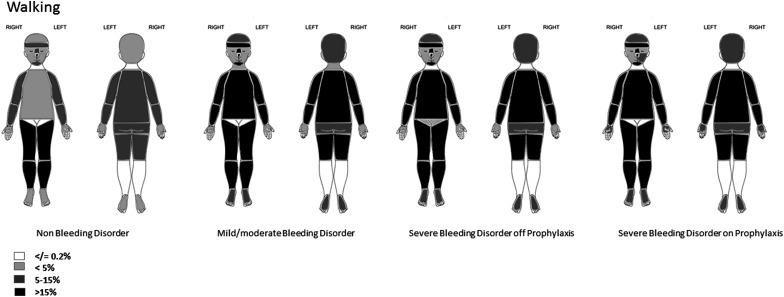
Location of bruises in walking children. The figure shows the proportion of collections in which there was at least one bruise at the location indicated.

Bruises on the cheeks, ears, neck, buttocks, eyes and genitalia were absent or extremely rare (<0.5% of collections) in children with bleeding disorders, regardless of severity, and absent in children without a bleeding disorder (table 4 and figures 1–3). Among children without a bleeding disorder and those with mild/moderate bleeding disorders, no more than 1% and 3% of collections, respectively, had a bruise in any other location. Children with severe bleeding disorders had substantially more collections with bruises (>10% of collections) predominantly on upper arms, feet, rear trunk, front of thighs and below knee.

**Table 4:** Percentage of collections with at least one bruise at the location indicated

	Premobile (number of collections)			Early mobile (number of collections)			Walking (number of collections)			
Location	Non-BD (1010)	Mild/moderate BD (59)	Severe BD (64)	Non-BD (479)	Mild/moderate BD (70)	Severe BD (64)	Non-BD (1082)	Mild/moderate BD (393)	Severe BD off prophylaxis (229)	Severe BD on prophylaxis (255)
Below knees	1	2	10	21	27	78	64	72	76	80
Left cheek	0	0	0	2	1	2	1	2	1	8
Right cheek	0	0	0	3	6	2	2	3	3	2
Left ear	0	0	0	0	0	3	0	1	0	0
Right ear	0	0	0	0	1	0	0	0	1	0
Head	1	3	2	7	11	6	3	8	6	11
Facial-T	1	0	3	14	20	16	11	18	16	19
Eyes	0	0	2	2	1	0	1	2	2	2
Front trunk	0	3	8	0	3	23	4	16	25	21
Rear trunk	1	0	11	2	3	16	10	21	17	17
Neck	0	0	0	0	1	0	0	1	0	0
Buttocks	0	0	0	0	9	4	5	10	6	8
Genitalia	0	0	0	0	0	3	0	0	2	0
Upper arms	0	2	19	1	11	34	9	27	30	33
Lower arms	0	0	5	1	16	39	12	21	28	30
Hands	0	0	2	1	0	13	1	3	3	8
Front thighs	1	2	10	4	23	23	17	27	21	30
Back thighs	0	0	6	1	4	11	7	17	16	19
Feet	0	2	14	1	3	9	4	6	13	9
Total	7	7	52	46	56	98	79	85	91	91

#### Early mobile children

Bruising was uncommon (≤3% of collections) across all groups on the ears, neck, eyes, front trunk, left cheek and genitalia. Bruising to the buttocks was recorded in bleeding disorders. Predominant sites of bruising in children without a bleeding disorder were below the knee and facial-T. The proportion of collections with bruising to the facial-T and head was similar across the three groups. In children with severe bleeding disorders, bruises were more frequent on the limbs, trunk, feet and hands than in the other two groups (with the exception of front of thighs in mild/moderate bleeding disorders).

#### Walking children

Bruises below the knee predominated, but were absent or reported in ≤3% of collections across all groups on the ears, right cheek, eyes, neck and genitalia; however bruises on the buttocks were recorded. Children with bleeding disorders had more bruises than those without bleeding disorders on the head, cheeks, front and rear trunk, upper and lower arms, facial-T, front and back of thighs, hands and feet. The proportion of collections with at least one bruise was similar for children with mild/moderate and severe bleeding disorders at most locations.

### Platelet disorders

One early mobile child with Glanzmann's thrombasthenia had 526 bruises in 12 collections: mean (range) 43.8 (29–72) bruises/collection. Mean size was 1.3 cm and the largest mean size was on the front and rear trunk. Bruises were very rare on the cheeks, hands, eyes, ears and genitalia although they were observed on the neck.

One child with hereditary macrothrombocytopenia contributed 3 premobile and 12 walking collections. No bruises were recorded when premobile. When walking there was a mean (range) 8.8 (1–16) bruises/collection. Bruises were not reported on the ears, cheeks, eyes neck, buttocks or genitalia. Most bruises were below the knee (75.5%) and facial-T (9.4%). Mean size was 0.94 cm and 67% of collections had at least one bruise >1 cm.

## Discussion

This study shows that children with bleeding disorders have more bruises than children without bleeding disorders and the difference is most marked at the premobile developmental stage. Children with severe bleeding disorders have significantly larger bruises compared with non-bleeding disorders at all mobility stages. The location of bruises differs between those with or without a bleeding disorder, but in all groups bruising on the ears, neck, cheeks, eyes or genitalia was rare. This is significant because bruising in these sites is indicative of physical abuse which should be investigated even in children with severe bleeding disorders.^9^,^18–20^

Bruising in premobile children is uncommon and warrants investigations for potential physical abuse.^6^ The fact that premobile children with a severe bleeding disorder had bruising in >50% of collections highlights the need for laboratory investigations for severe bleeding disorders in combination with child protection assessment. These findings may also explain why premobile children with undiagnosed severe bleeding disorders are commonly investigated for physical abuse.^1–4^ Our results suggest that a blood test compatible with a mild/moderate bleeding disorder cannot be assumed to be the cause of bruising in premobile children, because the proportion of collections with at least one bruise was the same as non-bleeding disorder children, and a rigorous assessment for possible abuse is required.

A key question addressed in this study is whether bruise location in children with bleeding disorders differs from those without a bleeding disorder. The small number of bruises in collections from premobile children without bleeding disorders or with mild/moderate bleeding disorders meant that no particular locations predominated in these groups. There were sites that were rarely bruised regardless of severity of bleeding disorder or mobility stage which include ears, neck, cheeks, eyes or genitalia.

Once children started to achieve independent mobility there were sites that were affected in all groups (below the knees, facial-T, head). These sites were predominantly on the front of the body and over bony prominences. The trunk, buttocks, limbs, feet and hands appeared to be more commonly affected in children with bleeding disorders than without. It is possible that bruises to the arms may result from handling young children with bleeding disorders during everyday care.

Surprisingly, the number and size of bruises in walking children with severe bleeding disorders did not vary with prophylaxis. It is possible that children on prophylaxis were allowed to be more active. Alternatively, children started on prophylaxis for haemarthroses may have been more prone to bruising, younger subjects may not have built up to full dose prophylaxis or insufficient numbers were investigated as prophylactic regimens vary between centres.

Study limitations include combining children with mild and moderate bleeding disorders because there were insufficient subjects to assess separately. It is likely that the bruise size and number would decrease as factor levels increased between moderately and mildly affected children and also within the mild group. There were insufficient subjects to investigate whether this was the case. Similarly there may have been unrecognised differences between type 1 and type 2 VWD. Bruises were reported by carers and so accuracy cannot be known with certainty. There was complete concordance between carer and researcher for bruise number and location when 40 collections from non-bleeding disorder children were assessed. The differences between carers' and researcher's estimates of bruise size were small, and it is unlikely that the differences in size could be entirely attributed to measurement errors; it is not known if the same pattern holds true for children with bleeding disorder. It is possible that parents of children with a bleeding disorder were more protective. If this was the case it would have had the tendency to reduce bruising and so the results reported may be underestimates. In all groups many bruises were recorded as exactly 1 cm. This is likely due to rounding and potentially introduces some inaccuracies when reporting bruises as >1 cm or <1 cm; further investigation is needed. The possibility that abuse occurred cannot be excluded, although the informed consent stated that any concerns would prompt a referral to child protective services. Two children had more bruises than expected and were reviewed by independent members of the local child protection team, and concerns regarding abuse excluded.

In conclusion, we show that bruises in premobile children with severe (but not mild/moderate) bleeding disorders are more common than in non-bleeding disorder children. Bruises of >1 cm are much more common in premobile children with severe bleeding disorders. Bruises on the ears, eyes, cheeks, neck and genitalia are rare, even among severe bleeding disorders. This is the first study to document bruise number, location and size in young children with bleeding disorders with a longitudinal design, and compare them with children without a bleeding disorder.
